# Supplementary material for: Umbilical Artery Thrombosis Masquerading as Single Umbilical Artery in a Stillbirth
Source: Diagnostics (Basel). 2025 Jan 3;15(1):94. doi: 10.3390/diagnostics15010094 (PMC11719595; doi:10.3390/diagnostics15010094)
Supplement: Supplementary file 1 [file diagnostics-15-00094-s001.zip › diagnostics-3218721-supplementary.pdf]

Supplementary Table S1: Clinicopathological Characteristics and Fetal Outcomes of Cases with Umbilical Artery Thrombosis

No.	Authors (Year)	No. of Cases (n)	Maternal Comorbid(s)/ Current/Previous Pregnancy Complications (n)	Prenatal diagnosis by USG (Yes/No)	GA at diagnosis, mean (range, weeks)	GA at delivery mean (range, weeks)	Mode of Delivery (n)	Umbilical Cord Abnormalities other than UAT (n)	Placental Histology (n)	Fetal Outcomes (n)
1	Nayak (1967)[10]	1	Uneventful	No	38weeks	38weeks	VD	Hypercoiling, stricture	Nucleated rbcs	Stillbirth
2	Devlieger et al. (1983)[11]	1	Uneventful	No	38weeks	38weeks	CS	Marginal insertion, short cord	No funisitis	Alive, fetal distress
3	Kristiansen and Nielsen (1985)[12]	2	Uneventful (1), history of stillbirth (1)	No	Term (2)	Term (2)	CS (1), VD (1)	Nuchal cord (1)	PI (2)	Stillbirth (2)
4	Heifetz (1988)[6]	20	Limited data available except polyhydramnios (1)	NA	NA	26 – 40weeks	NA	Acute funisitis (3), nuchal cord (5), marginal insertion (2), true knot (2), long cord (1), short cord (3), hypercoiling (4), stricture (3)	Acute chorioamnionitis (2)	Stillbirth (18), FGR (3), fetal distress (2), premature (1), adrenal vein thrombosis (1)
5	Cook (1995)[13]	1	Uneventful	Yes	31weeks+4 days	31weeks+4days	CS	Short cord	Numerous chorionic arteries emboli	Alive, fetal distress, preterm birth
6	Erkaya et al. (1999)[14]	1 (acardia twin)	NA	No	30weeks	30weeks	CS	Stricture	NA	Stillbirth
7	Byrd and Mayers (2000)[15]	1	Uneventful	No	39weeks+6 days	39weeks+6days	VD	No	Increase villous stromal fibrosis	Stillbirth
8	Larciprete et al. (2003)[16]	1	Uneventful	No	39weeks	39weeks	CS	No	Chorionic vasculitis, meconium laden macrophages	Alive, hypoxic lesion of basal nuclei and thalamus with severe tetraplegia

9	Sato and Benirschke (2005)[17]	11	High anticardiolipin titer (1)	NA	NA	36.8 (33 – 40weeks)	NA	Hypercoiling (2), peripheral cord insertion (3), long cord (3), short cord (2), acute funisitis (2), partial necrosis of vascular wall (11)	Mural thrombi (6), PI (4), chorangiosis (1), chorioamnionitis (2), FVM (2)	Stillbirth (2), FGR (3), fetal distress (1), proteinaemia (1), normal (4)
10	Klaritsch et al. (2008)[18]	1	IDDM, maternal blood group B negative	Yes	32weeks+4 days	34weeks+3days	CS	Hypercoiling	FVT	Alive, FGR
11	Avagliano et al. (2009)[19]	19	Uneventful (8), oligohydramnios (5), PPROM (2), preeclampsia (2), GDM (1), ANA+ (1), MTHFR homozygous for 677 polymorphism (1), active maternal HSV infection (1), maternal obesity (1), IDDM (1)	NA	NA	15 – 40weeks	NA	Cord prolapse (1), hypercoiling (2), marginal insertion (1), stricture (1), true knot (3), nuchal cord (2), Wharton jelly haemorrhage (1)	Necrotising deciduitis (1), PI (9), RPH (3), ATMCV (11), chorioamnionitis (3), VUE (3), MPFD (4), TSA (3), FTV (5), acute atherosis (1), HHPC (2), DVH (5), DVI (1), IH (2)	Normal (3), pneumonia (2), multivisceral haemorrhage (10), intrauterine infection (2), myocardial hypertrophy (3), intrahepatic UV thrombosis (1), fibroelastosis papillary muscles (3), cerebral oedema/haemorrhage (3), FGR (1), cardiac insufficiency (2)
12	Shilling et al. (2014)[20]	7	Uneventful (5), type 1 DM (1), Hepatitis B (1)	Yes (1)	NA	29 – 40weeks	NA	Marginal cord insertion (2), long cord (1), short cord (2), narrow cord (3)	UPI (3), FTV (4), nucleated RBCs (3), DVI (2)	Stillbirth (2), FGR (3), schizencephaly and partial acrania (1), caudate infarction (1)
13	Tanaka et al. (2014)[8]	2	NA	Yes	35weeks+5 days & 31weeks+3 days	36weeks+2days & 37weeks+4days	CS (2)	Hypercoiling (2), long cord (1)	UA thrombosis at chorionic plate (1)	Alive (2), FGR (2)
14	de Oliveira et al. (2016)[21]	1	Oligohydramnios	Yes	32weeks	34weeks	CS	Hypercoiling, long cord	NA	Alive, FGR

15	Alhousseini et al. (2018)[22]	1	Uneventful (1)	Yes	29weeks	33weeks	CS	UC medial myocytes necrosis	FVM, chorangiosis, PI	Alive, severe FGR, transient thrombophilia (severe protein S deficiency)
16	Lutfallah et al. (2018)[23]	1	Uneventful	Yes	33weeks+2 days	34weeks+3days	CS	Hypercoiling with necrosis	NA	Alive, preterm birth
17	Kitano et al. (2018)[24]	1	Uneventful	Yes	36weeks+4 days	37weeks	CS	No	Perivascular fibrin deposition and focal PI	Alive, severe fetal distress, therapeutic hypothermia HIE
18	Li et al. (2019)[25]	2	Uneventful (2)	Yes	36weeks+5 days & 38weeks	37weeks & 38weeks	CS (1), VD (1)	Hypercoiling (1)	NA	Alive (1), stillbirth (1)
19	Donepudi et al. (2019)[26]	1	Rhesus negative receiving intrauterine transfusion for fetal anaemia, GDM, mild polyhydramnios	Yes	28.1weeks	37weeks	CS	No	NA	Alive and well
20	Goto et al. (2020)[27]	1	Uneventful	Yes	33weeks+4 days	33weeks+5days	CS	Short cord, hypercoiling	NA	Alive, mild fetal distress
21	Zhu et al. (2020)[28]	3	Uneventful (1), history of stillbirth (1), GDM (1), thalassemia (1)	NA	NA	34weeks+3days – 39weeks	CS (1), VD (1)	Hypercoiling (1), excessive long (1), infarction (1)	NA	Stillbirth (1), preterm birth (1), normal (1)
22	Wei J et al. (2021)[29]	8	Uneventful (2), chronic nephritis with proteinuria (1), hypothyroidism (1), pre-eclampsia (1), Hepatitis B (1), *Listeria* infection (1), polyhydramnios (3)	Yes	24+ - 37+weeks	32+ - 37+weeks	CS (7), VD (1)	UC cyst (1), hypercoiling (3), true knot (1), velamentous cord insertion (1), no (4)	Chorioamnionitis (2), normal (5), small for GA (1)	All alive (8); fetal distress (2), sepsis (1), FGR (4), normal (2)
23	Zhang et al. (2021)[30]	1	Uneventful	No	31weeks	31weeks+2days	CS	Thin and narrow UC, velamentous cord insertion	NA	Alive, preterm birth
24	Wu et al. (2022)[31]	30	GDM (6), HDP (2), abnormal amniotic fluid (8)	Yes	28.9 – 36.2weeks	36.13 +/- 3.27weeks	CS (21), VD (8), NA (1)	Nuchal cord (15)	NA	Stillbirth (4), preterm birth (10), FGR (2), fetal distress (11)

25	Han et al. (2022)[32]	2	Uneventful (2)	Yes	36weeks & 33weeks+5 days	38weeks & 36weeks+1day	CS (2)	Hypercoiling (1)	NA	Both alive (2)
26	Li et al. (2023)[33]	2	GDM (1), ANA+ (2), Hepatitis B carrier (1)	Yes	27weeks+1 day & 30weeks+4 days	34weeks+6days & 31weeks+1day	CS (2)	Short cord (1), hypercoiling (1), nuchal cord (1)	DVI (1), villous oedema (1), mild acute subchorionitis (1)	Both alive (2), preterm birth (2)
27	Dindinger et al. (2023)[34]	1	Multiple DVT on heparin, IVC filter and venous stent placement complicated with HIT, HDP, GDM	Yes	27weeks	37weeks	CS	No	Normal	Alive, mild respiratory distress
28	Wang et al. (2023)[9]	10	Uneventful (6), hypothyroidism (1), Hepatitis B (1), myoma (1), congenital heart disease (1), HDP and GDM (1)	Yes	29.9 +/- 3.7 (23 – 31weeks+6 days)	36.3 +/- 2.5 (31weeks+6days – 38weeks+6days)	CS (9), VD (1)	Nuchal cord (2), hypercoiling (7), stricture (1), battledore cord insertion (2), no (3)	NA	All alive (10), FGR (6), fetal distress (2), congenital anomalies (3), neonatal hypoglycaemia (1), neonatal thrombocytopaenia (1), normal (2)
29	Wang et al. (2023)[35]	2 (MCDA twin)	PPROM	Yes	29weeks+5 days	32weeks	CS	Marginal cord insertion (2), hypercoiling (1)	NA	Alive (2), preterm birth (2)
30	Liu et al. (2023)[36]	1 (one DCDA twin)	Uneventful	Yes	35weeks+6 days	35weeks+6days	CS	Hypercoiling	Mild chorioamnionitis, PI	Alive, preterm birth
31	Pan et al. (2024)[37]	46	Thrombophilia (3), GDM (8), gestational hypothyroidism (11), HDP (6), abnormal amniotic fluid (12)	Yes	29.71 (22.96 – 34.29weeks)	37.07 +/- 3.134weeks	CS (29), VD (17)	Abnormal cord insertion (8)	NA	Stillbirth (1), preterm birth (20), FGR (18), macrosomia (2), fetal distress (10), intraamniotic infection (17)
32	Tu et al. (2024)[38]	76	GDM (17), HDP (11), chronic thyroid disease (5), coagulation disorders (3), abnormal amniotic fluid (12)	Yes	30.6 (25 – 35.2weeks)	36.1 (32.2 – 38.1weeks)	CS (59), VD (17)	Hypercoiling (28)	NA	Stillbirth (7), FGR (14), NICU admission (10)

33	Hong et al. (2024)[39]	19	NA	Yes	28weeks+4 days–39weeks+1 day	31 – 39weeks+5 days	CS (18), defaulted (1)	Hypercoiling (15), pseudoknot (1)	NA	FGR (9), fetal distress (2), preterm birth (10)
34	Gong et al. (2024)[40]	1	Antiphospholipid syndrome – treated with S/C LMWH calcium from 23+4 weeks GA	Yes	31weeks	31weeks+3days	CS	Dull red, rigid UC	NA	Alive, FGR, preterm birth
35	Jin and Zhang (2024)[41]	1	Uneventful	Yes	30weeks+1 day	30weeks+1day	VD	Wharton jelly congested and oedematous, UC segmental vascular wall infarction	Severe chorioamnionitis	Alive, fetal distress, preterm birth
36	Gladstone et al. (2024)[42]	1	Uneventful	Yes	36weeks+1 day	36weeks+3days	VD	NA	High grade FVM	Alive, FGR
37	Romani et al. (2024)[7]	1	Uneventful	Yes	33weeks+5 days	33weeks+5days	CS	Hypocoiling, many strictures	PI	Alive, preterm birth
38	Li et al. (2024)[43]	1	Uneventful	No	39weeks	39weeks	VD	Irregular coiling (borderline hypercoiling intermixed with hypocoiling)	Acute and chronic chorioamnionitis, small calcification	Alive and well
39	Current case	1	Uneventful	No	24weeks	24weeks	VD	Hypercoiling	FVM	Stillbirth

Abbreviations: ANA – antinuclear antibody; ATMCV – acute thrombosis of major chorionic vessels; CS – caesarean section; DVH – distal villous hypoplasia; DVI – distal villous immaturity; DVT – deep vein thrombosis; FGR – fetal growth restriction; FTV – fetal thrombotic vasculopathy; FVM – fetal vascular malperfusion; GA – gestational age; GDM – gestational diabetes mellitus; HDP – hypertensive disorders of pregnancy; HHPC – histologic hypoxic placental changes; HIE – hypoxic ischaemic encephalopathy; HIT – heparin-induced thrombocytopaenia; HSV – herpes simplex virus; IDDM – insulin-dependent diabetes mellitus; IH – intraplacental haematoma; IVC – inferior vena cava; MPFD – massive perivillous fibrin deposition; n = number of cases; NA – not available; NICU – neonatal intensive care unit; PI – placental infarction; PPROM – preterm premature rupture of membrane; rbcs – red blood cells; RPH – retroplacental haematoma; S/C LMWH – subcutaneous low molecular weight heparin; TSA – thrombosis of spiral artery; UAT – umbilical artery thrombosis; UC – umbilical cord; USG – ultrasound; UV – umbilical vein; UPI – uteroplacental insufficiency; VD – vaginal delivery; VUE – villitis of unknown etiology; Y/N – yes/no
